# Proteolytic Enzymes Clustered in Specialized Plasma-Membrane Domains Drive Endothelial Cells’ Migration

**DOI:** 10.1371/journal.pone.0154709

**Published:** 2016-05-06

**Authors:** Monica Salamone, Francesco Carfì Pavia, Giulio Ghersi

**Affiliations:** 1 IAMC-CNR, U.O. Capo Granitola, Mazara del Vallo (Trapani), Italy; 2 Dipartimento di Ingegneria Civile, Ambientale, Aerospaziale, dei Materiali; Università di Palermo,Viale delle Scienze, ed. 6 -90128 Palermo, Italy; 3 Dipartimento di Scienze e Tecnologie Biologiche, Chimiche e Farmaceutiche, Università di Palermo, Viale delle Scienze, ed. 16 -90128 Palermo, Italy; Stony Brook University, UNITED STATES

## Abstract

*In vitro* cultured endothelial cells forming a continuous monolayer establish stable cell-cell contacts and acquire a “resting” phenotype; on the other hand, when growing in sparse conditions these cells acquire a migratory phenotype and invade the empty area of the culture. Culturing cells in different conditions, we compared expression and clustering of proteolytic enzymes in cells having migratory versus stationary behavior. In order to observe resting and migrating cells in the same microscopic field, a continuous cell monolayer was wounded. Increased expression of proteolytic enzymes was evident in cell membranes of migrating cells especially at sprouting sites and in shed membrane vesicles. Gelatin zymography and western blotting analyses confirmed that in migrating cells, expression of membrane-bound and of vesicle-associated proteolytic enzymes are increased. The enzymes concerned include MMP-2, MMP-9, MT1-MMP, seprase, DPP4 (DiPeptidyl Peptidase 4) and uPA. Shed membrane vesicles were shown to exert degradative activity on ECM components and produce substrates facilitating cell migration. Vesicles shed by migrating cells degraded ECM components at an increased rate; as a result their effect on cell migration was amplified. Inhibiting either Matrix Metallo Proteases (MMPs) or Serine Integral Membrane Peptidases (SIMPs) caused a decrease in the stimulatory effect of vesicles, inhibiting the spontaneous migratory activity of cells; a similar result was also obtained when a monoclonal antibody acting on DPP4 was tested. We conclude that proteolytic enzymes have a synergistic stimulatory effect on cell migration and that their clustering probably facilitates the proteolytic activation cascades needed to produce maximal degradative activity on cell substrates during the angiogenic process.

## Introduction

Angiogenesis is a fundamental process in vascular remodeling during embryogenesis as well as in wound healing in adults. Moreover, in several pathological conditions such as rheumatoid arthritis, diabetic retinopathy, psoriasis, hemangiomas, and cancer, atypical angiogenesis is observed. Since angiogenesis involves migration/invasion of endothelial cells through connective tissues, proteolytic enzymes play a non-secondary role in the process.

The proteases involved generally belong to the extracellular matrix metalloproteinase (MMP) [1–4] and to the serine protease [5–7] families. Some proteases which belong to these families have also been observed as being targeted by adhesion molecules such as α_v_β_3_ [8, 9] and α_3_β_1_ [10–15] to specific plasma membrane domains (invadopodia-like structures) where they promote cell migration and invasion into ECM.

Expression of several MMPs (interstitial collagenases, gelatinases and MT-MMPs) in endothelial cells is induced by VEGF [16, 17] and their activity is controlled by specific inhibitors, the tissue inhibitors of metalloproteases (TIMPs), that act on catalytic sites of MMPs [18]. TIMP-2 and TIMP-4 for instance were shown to inhibit tubulogenesis induced by VEGF/FGF-2 growth factors, while other MMPs inhibitors including TIMP-1 had no effect on this phenomenon [18]. Trans-membrane proteolytic enzymes, in particular MT1-MMP, were also shown to be highly involved in invasion mechanisms [19]. In endothelial cells with migratory phenotype, it has been demonstrated that MT1-MMP is over-expressed [20, 21]. Furthermore, in others experimental systems, it was established that MT1-MMP over-expression resulted in localizing this protease in invadopodia, where it initiated a proteolytic cascade leading to cell invasion [22, 23].

Proteolytic enzymes belonging to serine protease family, and type-II transmembrane serine proteases (TTSPs) in particular, including dipeptidyl peptidase 4 (DPP4/CD26) and seprase/fibroblast activation protein alpha (FAP-α), are thought to increase the pro-invasive properties of MMPs and integrins [24, 25]. DPP4 and seprase are not expressed on the cell surface of differentiated endothelial and stroma cells, but they are located on the cell surface of invasive cancer cells and on the surface of endothelial cells while wounds are healing [12, 20, 26]. Once wounds have healed, DPP4 is re-targeted to membrane sites facing the basement membrane, supporting both its role in degrading collagenous matrices, and as an adhesion molecule [27–28].

Endothelial cells forming new vessels and invasive tumor cells share several similarities; however, whereas tumor cells are abnormal, uncontrolled cells showing unusual behavior, endothelial cells are normal and their behavior is under the control of specific molecular mechanisms. Moreover, in vitro, endothelial cells can be induced to assume an invasive phenotype by cell culture conditions. They represent, therefore, an excellent model with which to analyze invasion mechanisms by comparing invasive and non invasive cells with the same genetic background.

Tumor cells have been shown to invade ECM by extending specialized plasma membrane protrusions (invadopodia) enriched in proteolytic enzymes [29]. Moreover, invasive tumor cells were also shown to release in the extracellular space membrane vesicles [30, 31], originating from specialized plasma membrane domains. It has been suggested that vesicles play a role in cell migration and tumor invasion [32] and several proteases associated with these structures have been identified [33].

Vesicle shedding is a living phenomenon modulated by extra-cellular signaling [34] and is morphologically similar to virus budding [35, 36]. It occurs both *in vivo* and *in vitro* involving cell-cell and cell-matrix interaction mechanisms equally important in both tumor and normal cells [33].

As shown by Taraboletti et al. (2002), when human umbilical vein endothelial cells (HUVEC) were treated with angiogenetic factors such as fibroblast growth factor 2 (FGF-2), vascular endothelial growth factor (VEGF), and Thrombospodin-1, there was also an increase in the release of vesicles and associated proteases [37]. Shed vesicles and specialized plasma membrane protrusions therefore appear to play a comparable or complementary role in both endothelial and tumor cell invasion mechanisms.

In this study we analyzed the profiles of major serine and metallo proteases expressed both on plasma membrane domains and in shed membrane vesicles when endothelial cells acquire an “invasive” phenotype. We tested their role in degrading ECM components and consequentially in driving endothelial cell migration by generating permissive substrates.

## Experimental Procedures

### Cells

ECV-304 (IZSBS BS CL 137), a continuous human endothelial cell line, was purchased from the “Istituto Zooprofilattico” in Brescia, Italy. The endothelial cells were cultured in Medium 199 (GIBCO, Grand Island, NY) supplemented with 2 mM L-glutamine, 1 unit/ml penicillin, 10 μg/ml streptomycin, 5 units/ml heparin, plus 10% fetal bovine sera and H_2_CO_3_ 50 mM pH 7.4. We have cultured ECV-304 cells in two conditions: confluence (C), ECV-304 cells reach stable cell-cell contacts into a 75 cm^2^ culture plate and migrating (M), ECV-304 cells coming from one plate of confluence cell (75 cm^2^) were cultured into a 175 cm^2^ culture plate. Cell extracts, plasma membrane and shed vesicles were obtained after removing regular media, three washes in complete PBS (containing Ca^++^ and Mg^++^ ions), after 3 hr incubation in serum free media. Quantification of different kind of samples were normalized to 100% corresponding specifically to one 75 cm^2^ plate for confluence cells (C) and one 175 cm^2^ plate for migrating cells (M).

ECV-304 cell motility was evaluated as reported [38] “S1 File”.

### Antibodies

Rat mAbs, E19, directed against human placental DPP4, rat mAbs D8, against human placental seprase [13, 39], rat mAb C27 against human melanoma β1 integrin [10] were produced in the lab of W.T. Chen; rat mAb 13 against MT1-MMP was produced in the Palermo laboratory and selected by western blotting and immunolocalization in KKLS cells over expressing the analyzed molecule. Goat anti-uPA (C-20) was purchased from Santa Cruz Biotechnology, Inc. Rabbit, anti-membrane-type matrix metallo-proteinase-1 hinge region, anti-matrix metalloproteinase-2, anti-matrix metalloproteinase-9 and anti TIMP-2 polyclonal antibodies and mouse mAb anti-α-smooth muscle actin clone 1A4 FITC conjugate were purchased from Sigma, US. Mouse mAb against Fibronectin from Usbiological, US, mouse mAb against Filamin clone PM6/317 from Serotec, US.

### FEI Quanta 200 FEG MKII scanning electron microscope analyses

For Scanning Electron Microscopy (SEM) observation the ECV-304 cells, were grown onto glass slide; when confluence was reached wonding was performed as reported in “**Cell Migration Assay**”, cells were washed with fresh PBS containing Ca^++^ and Mg^++^, fixed with paraphormaldeide 3,7% in PBS containing Ca^++^ and Mg^++^, dehydrated with ethanol series (25%, 50%, 75% v/v and pure ethanol) and finally dried at room temperature. The as prepared cells were observed by using a SEM-FEI QUANTA 200FEG. The glasses were first placed onto a conductive stub and then gold sputtered (Sputtering Scancoat Six, Edwards) for 40 s under argon atmosphere before imaging. SEM images were exported for further analysis.

#### Shed membrane vesicles purification from conditioned medium

Vesicles were purified from a conditioned medium of endothelial cells as described [34]. In brief, a medium conditioned for 3 hrs by sub-confluent (migrating) or confluent (confluent) healthy cells was centrifuged at 2,000 x g and at 4,000 x g for 15 min. The supernatant was ultra-centrifuged at 105,000 g for 90 min. Pellected vesicles were resumed in PBS. The amount of isolated vesicles was determined by measuring protein concentration using the Bradford microassay method (Bio-Rad) with bovine serum albumin (Sigma) as a standard.

### Plasma membrane purification

Cells were cultured to low concentration to prevent cell-cell contact formation (Migration) or confluence (Confluence) in 175 cm^2^ flasks in complete media. 5 x 10^7^ cells were collected by centrifugation at 1,500 rpm/5’, resumed in 20 vol of PBS (weight/volume) and homogenized in a small clearance pestle—dounce homogenizer and centrifuged to 15,000 rpm X 10’ in a Beckman J-20 centrifuge. The pellet was resumed with a syringe in 45 vol of PBS, loaded on a 48–60% sucrose gradient and centrifuged to 20,000 rpm x 16 h 30’ at 4°C in a Spinco Beckman SW25 rotor. Gradient interfaces were recovered using a syringe and diluted 5 times in H_2_O, than centrifuged to 30,000 rpm X 2 h in a Spinco Ti50 rotor. The pellet was resumed in a two phase system, 40 ml constituted by dextran and polyethylene glycole, centrifuged to 5.000 rpm 150’ in a Beckman J13 rotor. Purified membranes at the interface were collected with a syringe in 50 ml H_2_O, and centrifuged at 50,000 rpm X 1 h at 4°C in a Spinco Beckman Ti50. Purified membranes were used in western blotting and zymography procedures.

### Triton X-100 extract from cell bodies, membranes and shed membrane vesicles

Cells were cultured in 175 cm^2^ dishes to confluence or sub-confluence and were washed three times with 25 ml PBS 5’, 4°C. Cell bodies, as well as partially purified plasma membranes and shed membrane vesicles were extracted with 0.1% Triton X-100 (Sigma) in PBS. Extractions were performed for 3 hours at 4°C. Extracts were clarified by an initial centrifugation at 2,000 rpm/10’ at 4°C, followed by a second one at 10,000 rpm/15’ at 4°C. Samples were concentrated using Millipore filters Ultrafree-15 PBTK (Bedford, MA) to 1 mg/ml and used in immunoblotting, gelatin zymography and DPP4 substrate overlay assays.

### Gelatin zymography assay

Isolated proteins from cell bodies, partially purified plasma membranes and shed membrane vesicles were analysed by gelatin zymography performad as described by Pineiro-Sanchez et al [13].In particular, gelatin zymographies were incubated 48 hrs at 37°C into different developing buffers: 1) Tris-HCl buffer 50 mM pH 7.4 containing 1.5% Triton X-100 and 0.02% Na Azide plus 2 mM CaCl_2_, to detect both serine that matrix metallo-proteinases; 2) Tris-HCl buffer 50 mM pH 7.4 containing 1.5% Triton X-100 and 0.02% Na Azide plus 2 mM EDTA, to detect only serine proteinases.

### Immunostaining of endothelial cells grown in 2D and 3D systems

For assays in 2D systems, endothelial cells were seeded inside 12 well plates on glass covers (diameter 18 mm) which had been coated with 50 μg/ml rat tail type-I collagen in 0,02N acetic acid (2h. r.t.) and neutralized by the addition of complete media containing 10% fetal calf serum (0,5 ml per wash). When cells reached confluence a wounding edge was performed as later described in this section (Cell migration assays). After different times of recovery, cells were fixed for 3 min. in 3% phormaldeide in PBS, and processed with specific antibodies in indirect fluorescence staining [40].

For assays in 3D systems, endothelial cells were cultured inside type I collagen gels and processed in direct immunofluorescence experiments as previously described [20].

### Two-dimensional zymography analyses

Two different approaches were used to discriminate proteins in first dimension.

A) Shed membrane vesicle proteins extracted as previously described were applied to Immobiline^™^ pH 3–10 (18 cm—Amersham Biosciences, Sweden) and were run according the manufacturer’s instructions, (the amount of protein utilized was 67 μg/strip). The sample lanes were equilibrated in Laemmli’s buffer [41] in which no reducing reagent or SDS was present. Second-dimension gel was prepared as a 9–16% SDS-PAGE gelatin zymography gradient. The equilibrated piece of gels (Immobiline^™^) were placed on the second-dimension and separated under non-reducing conditions. After electrophoretic separation, gels were washed three time in 2.5% Triton X-100 plus 0.02% Na Azide in H_2_O, then incubated 48 hrs at 37°C under the following conditions: a) Tris-HCl buffer 50 mM pH 7.4 containing 1.5% Triton X-100, 2 mM CaCl_2_ and Na Azide 0.02%; b) in Tris-HCl buffer 50 mM pH 7.4 containing 1.5% Triton X-100, 2 mM CaCl_2_, 50 nM AEBSF, 2 mM PMFS and Na Azide 0.02%; c) in Tris-HCl buffer 50 mM pH 7.4 containing 1.5% Triton X-100, 2 mM CaCl_2_, 10 μM E64 and Na Azide 0.02%. ProMMP-2 and proMPP-9 from human blood plasma were used as markers.

B) Proteins extracted from purified plasma membranes (40 μg/well) and from shed membrane vesicles (10 μg/well) were applied on a 6% acrylamide gel containing 9M urea (BioRad, Hercules, CA) and ampholine pH 5.0–8.0 (Pharmacia LKB, Biotechnology AB), which were run at 4°C for 3 h at a constant voltage (400V). The sample lanes were cut out and equilibrated in Laemmli’s buffer [41] in which no reducing reagent or SDS was present. The equilibrated pieces of gel were placed on the second-dimension and separated under non-reducing conditions on 7.5% SDS-PAGE gelatin zymographies. After protein separation, the gelatin zymographies were washed and incubated in specific buffers as previously described.

### Shed membrane vesicle immunostaining

Shed membrane vesicles, obtained as previously described from confluence and migrating ECV-304-conditioned medium cells, were seeded on glass coated with a 1% polylisine solution in PBS. The glasses were washed to remove any vesicle excess and blocked with 3% BSA in PBS for 2 hrs; than washed 3 times in PBS and incubated with primary antibodies for 2 hrs. After that the glasses were washed 3 times with PBS and treated with secondary antibodies, against the specific species of primary, conjugated to FITC, TRITC or Texas Red fluorocromes and incubated 1 hr. At this point, the glasses were washed 3 times with PBS and mounted with mounting solution [80% glycerol in tris 0.1 M pH 7.4 plus NaN_3_ 0.02% (c.f.)].

### Labeling of Laminin-V, type-I collagen and type-IV collagen fibers

Type-I Collagen was polymerized prior to biotin labeling so that sites of polymerization were not perturbed; collagen type-IV and Laminin-V were biotin labeled while in solution. Specifically, 10 ml of collagen type-I solution (rat-tail collagen type-I, 4.66 mg/ml, Collaborative Biomedical Products, Becton and Dickinson Labware, Bedford, MA) were mixed with 10-ml DMEM at 4°C. The mixture was incubated for 30 min at 37°C to allow polymerization of the collagen. The gel was washed with 30 ml of coupling borate buffer, pH 9.3 (Sigma) for 30 min and then incubated with 30 ml borate buffer containing 3 mg of Sulfo-NSH-Biotin (Pierce) at 25°C on a shaker. Conjugation was stopped by washing 3 times with PBS, followed by a 50 ml PBS washing for 2 days and a 50 ml distilled water wash for another 2 days. Labeled collagen fibers were solubilized in acidic water (0.02N acetic acid) to a final concentration of 1 mg/ml. Labeled collagen monomers were mixed with equal volumes of p-buffer (300 mM NaCl in 50 mM ammonium bicarbonate buffer, pH 8.4) and incubated for 30 min at 37°C to allow gel formation. Type-IV collagen and Laminin-V were resumed at 2 mg/ml in a coupling buffer (50 mM borate buffer pH 9.3) conjugated 30’ with 75 μg of Sulfo-NHS-Biotin at 25°C on a shaker. The samples were extensively dialyzed against PBS to stop the conjugation reaction and to remove non-linked biotin.

### ECM degradation assays

Laminin V and type-IV collagen, biotinated as described above, and non-labeled Fibronectin (50 μg/sample) were incubated in presence of membrane vesicles (20 μg) shed by endothelial cells cultured to confluence (C) or in migration (M) conditions. The degradative activity of enzymes on type-I collagen fibril gels was measured by the release of collagen peptides from the biotinylated type-I collagen gel. To prepare a biotinylated collagen gel as a protease substrate, 10 μl biotinylated type-I collagen solution (1 mg/ml) was mixed with 10 μl p-buffer, and incubated for 1 h at 37°C to allow gel formation. The collagen gel was washed with PBS, and 20 μl of shed membrane vesicles (containing 100 μg of proteins) coming from endothelial cells cultured in active migration or to confluence were added. MMP-2 (recombinant form isolated from COS-1 MMP-2 transfectant), was applied at the concentration of 50 ng/ml and used as a control for biotinylated peptide release. Incubations were performed at 37°C for 15’–48 hrs. The supernatant could contain auto-released collagenous peptides and was accessible to MMP-2 digestion (data not shown). It showed obvious denatured collagen; thus, the collagen gel in this study is referred as “gelatin” throughout the text. The reaction was stopped by low speed centrifugation (2,000 rpm) at 4°C for 10 min. Resulting supernatants containing biotinylated gelatin peptides were solubilized with a 2X SDS sample buffer and analyzed by SDS-PAGE (7.5% gel). Blots containing biotinylated gelatin peptides were stained with HPR conjugated streptavidin and the ECL system (Amersham). Fibronectin peptides were analyzed by immunoblotting using mAb against Fibronectin (Usbiological) and stained with HPR conjugated streptavidin and the ECL system (Amersham).

### Cell migration assays

Monolayer wound cultures (see Immunostaining of endothelial cells grown in 2D and 3D systems) overlaid by Collagen type-I fibers with or without shed membrane vesicles were used to examine cell migration in collagen type-I gels during wound closure. ECV-304 were grown to confluence in 2-well chambered coverslips (Lab-Tek, Rochester, NY). The monolayer was scratched with a pipette tip to generate wound edges. Culture media was then replaced with collagen type-I fibrils in culture media (600 μg/ml; 50 μl/well) either without or with different concentrations of shed membrane vesicles shed by ECV-304 cells cultured to confluence (-) or in migrating (+) conditions. Cultures were allowed to gel in a CO_2_ incubator for 30 min at 37°C. Media were added containing either one of the following or no proteases inhibitors: AEBSF 20 μM or CT1847 50 nM. Effects on cell migration were observed in real time using phase contrast microscopy (Leika inverted Microscope). Cell migration was quantified by measuring the wounding closure area by migratory cells, using NIH Image 1.62b4/fat analysis program. Each block of data was valued to statistical significance by P value, this was in all experiment minus of 0,01.

## Results

### ECV-304 motility and immunolocalization of proteolytic enzymes in cells cultured in 2D and 3D systems

**Fig 1:**
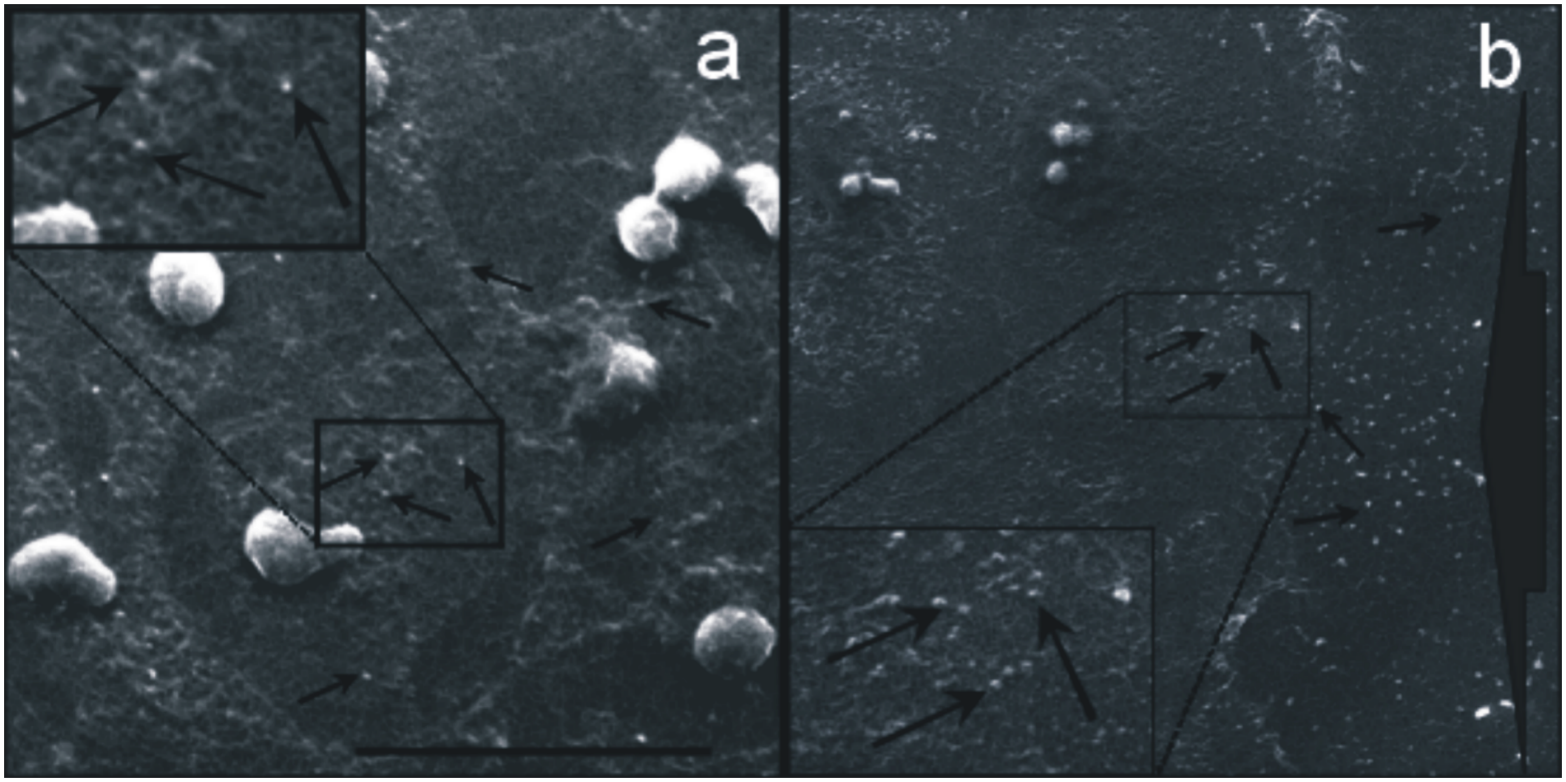
Scanning Electromicroscope Images. ECV-304 cells cultured to confluence (a) and at the wound hedge border (b) were analyzed by SEM. In the figure arrows indicate shed membrane vesicles; the squared area were magnified 2X. The bar = 40 μm.

ECV-304 cells cultured in confluence “Fig 1a” and migration “Fig 1b” were analyze by SEM analyses about release of shed membrane vesicles. As shows in “Fig 1” ECV-304 cells release shed membrane vesicles in both cell conditions.

**Fig 2:**
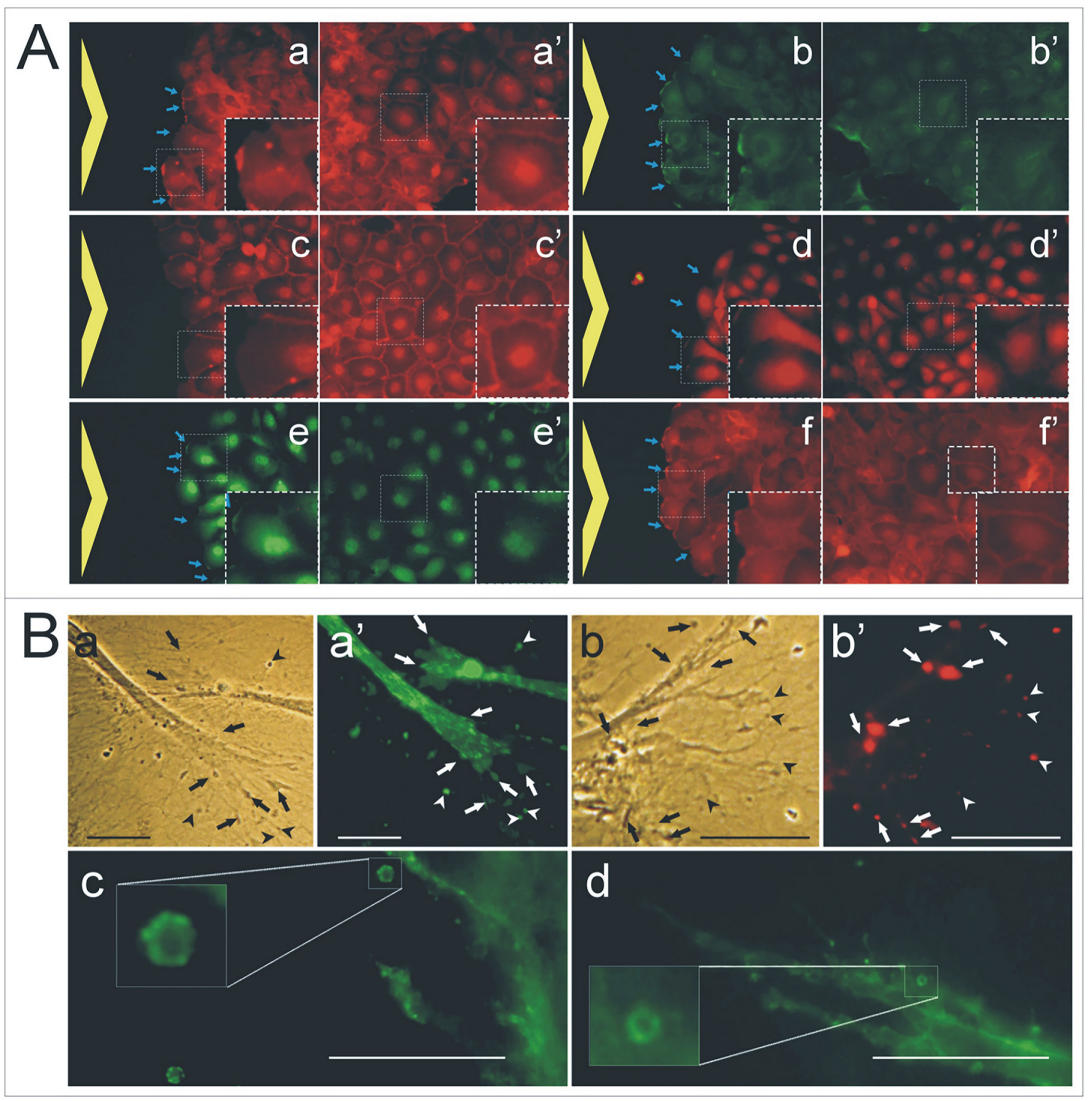
Immunostaining of endothelial cells cultured in 2D wounded and in 3D type-I collagen fibril gel systems. **(A)**—Endothelial cells were cultured to confluence and wounded. After 4 hours, cells were fixed and analyzed in immunofluorescence with antibodies against interest molecules; (a–f) cell staining of cells invading the wound healing area, (a’–f’) cell staining of corresponding unwounded areas, in which cells form a mono-layer. In (a–a’) anti MT1-MMP distribution; in (b–b’) anti MMP-2 distribution; in (c–c’) anti β-catenin distribution; in (d–d’) anti DPP-4 distribution; in (e–e’) anti seprase distribution; in (f–f’) anti β_1_-integrin distribution. Yellow arrows show the wound area; while the blue arrows indicate immunostaining at the migration front of cells invading the wound healing area. Each squared area represents a 2X magnification to better mark, or not mark, immunolocalization of specific antigens in migrating and non-migrating cells. Barr = 50 μm. **(B)**–Endothelia cells were cultured inside to 3D type-I collagen fibril gels. (a–a’) phase contrast and anti DPP-4-FITC conjugate staining, respectively; (b–b’) phase contrast and anti MT1-MMP-TRITC directly conjugate staining, respectively; (c) anti seprase staining; (d) anti β_1_-integrin staining; in c and d, the squared areas are 4X magnification of shed membrane vesicles released inside to type-I collagen fibril gel networks. In the images, the arrows indicate cell plasma membrane protrusions, and arrowed shed membrane vesicles released by cells in the extracellular matrix. Bar = 5 μm.

To analyze whether the expression of proteolytic enzymes in specialized domains of plasma membranes occurred at a more pronounced level when endothelial cells acquired a migratory phenotype, we cultured cells directly on cover glasses coated with type-I collagen fibril substrate and, when confluence was reached, we “wounded” them as described in experimental procedures. In the same sample, migrating cells at the healing wound as well as cells at confluence in the non-wounded area were stained with antibodies against different proteolytic enzymes. The results of these experiments are illustrated in “Fig 2A”. MT1-MMP is observed to localize on plasma membrane protrusions of cells invading the wound area “Fig 2Aa”, as well as at the level of plasma membranes of cells forming stable cell-cell contacts at the level of the continuous mono-layer “Fig 2Aa’”; MMP-2 was observed on plasma membranes of cells at the wound healing boundaries “Fig 2Ab”, while a very faint and non continuous localization of the protein was detected in the plasma membrane of cells at the continuous mono-layer “Fig 2Ab’”. As positive control of plasma membrane staining in cells forming cell-cell contacts, we also used a mAb against β-catenin. As show in “Fig 2Ac’”, all plasma membranes of cells forming cell-cell contacts were very well stained; however, at the front of the wound area “Fig 2Ac” no staining was detectable in the plasma membranes of invading cells. Antibodies against DPP-4 “Fig 2Ad” and seprase “Fig 2Ae” recognized their specific antigens on the plasma membrane of cells invading the wound healing area, but any staining was observed on the plasma membrane of cells forming stable cell-cell contacts “Fig 2Ad’ and 2Ae’, respectively”. A different picture was observed using mAb C27 against β_1_-integrin, in this case “Fig 2Af and 2Af’” immunolocalization on plasma membrane(s) of both migrating and confluent cells was detected.

To visualize proteolytic enzymes in shed membrane vesicles, we cultured endothelial cells in a 3D type-I collagen fibril gel. In this culture condition, shed membrane vesicles are blocked inside the collagen fibril network of the gel and they can be visualized by phase-contrast microscopy and tested in immunostaining experiments “Fig 2B”. Cell and shed membrane vesicles were analyzed with antibodies against different molecules. Antibodies against DPP-4 (Ba), MT1-MMP (Bb), seprase (Bc) and β_1_-integrin (Bd) stained shed membrane vesicles.

### Shed membrane vesicles production from ECV-304 and immunostaining

**Fig 3:**
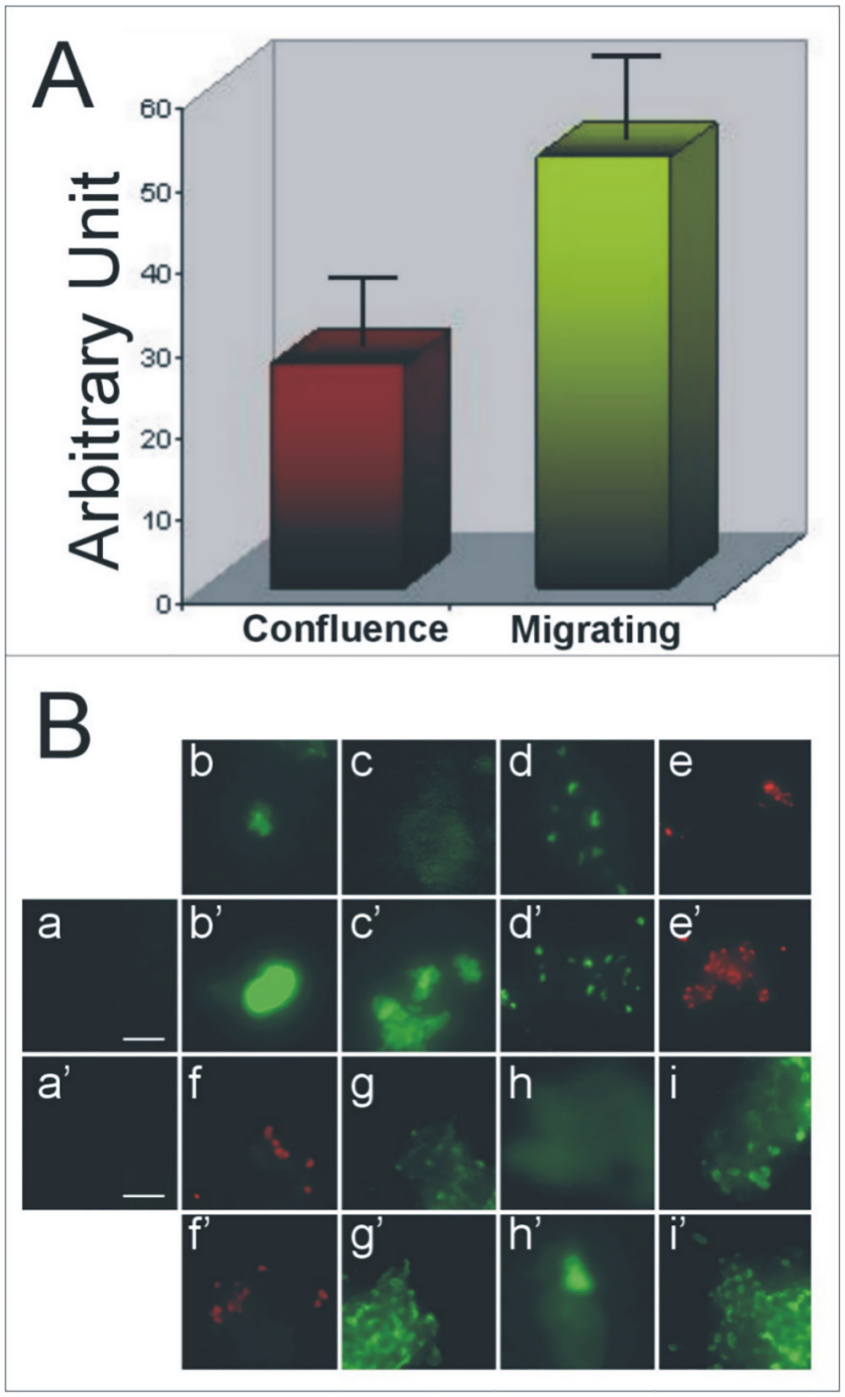
Evaluation of shed membrane vesicles released from ECV-304 cultured to confluence and in migrating conditions; immunodetection of antigens by immunofluorescence staining. **(A)**—Shed membrane vesicles released from the same amount of ECV-304 cultured to confluence or in migrating conditions were compared to the amount of total proteins using Bradford colorimetric methods and reported as Arbitrary Units in the ordinate. **(B)—**Shed membrane vesicles obtained from confluent (a-i) or migrating (a’-i’) ECV-304 were coated on polylisine-treated cover glasses and immunostained with different primary antibodies against: (b-b’) mAb C27 against β_1_-integrin; (c-c’) mAb D28 against seprase; (d-d’) mAb E34 against DPP4; (e-e’) anti MMP-2 (Sigma); (f-f’) mAb 13 against MT1-MMP; (g-g’) anti MMP-9 (Sigma); (h-h’) anti UpA (Santa Cruz Biotechnology); (i-i’) anti TIMP-2 (Sigma). The immunoreactions were detected using secondary antibodies against the primary animal species conjugated with FITC or TRITC fluorochrome; in (a-a’) vesicles were treated with a mixture of fluorescence conjugated secondary antibodies. The reported staining is not a quantitative evaluation of antigens. Bar = 2 μm.

Membrane vesicles are produced by endothelial cells when they have well-differentiated epithelia phenotypes such as when they acquire a mesenchymal phenotype. As shown in “Fig 3A”, the amount of shed membrane vesicles released increases in migrating mesenchymal ECV-304 which produced about two times the number of vesicles compared to confluent ones. Moreover, shed membrane vesicles from ECV-304 cells cultured into the two different morphological conditions, to confluence or migrating, were analyzed by immunostaining for the presence of several proteolytic enzymes such as related molecules; as shown in “Fig 3B”, analyzed molecules were detected both in confluence and migrating vesicles types.

### Gelatin zymography and immunoblotting assays to detect proteolytic enzymes in endothelial cell compartments

**Fig 4:**
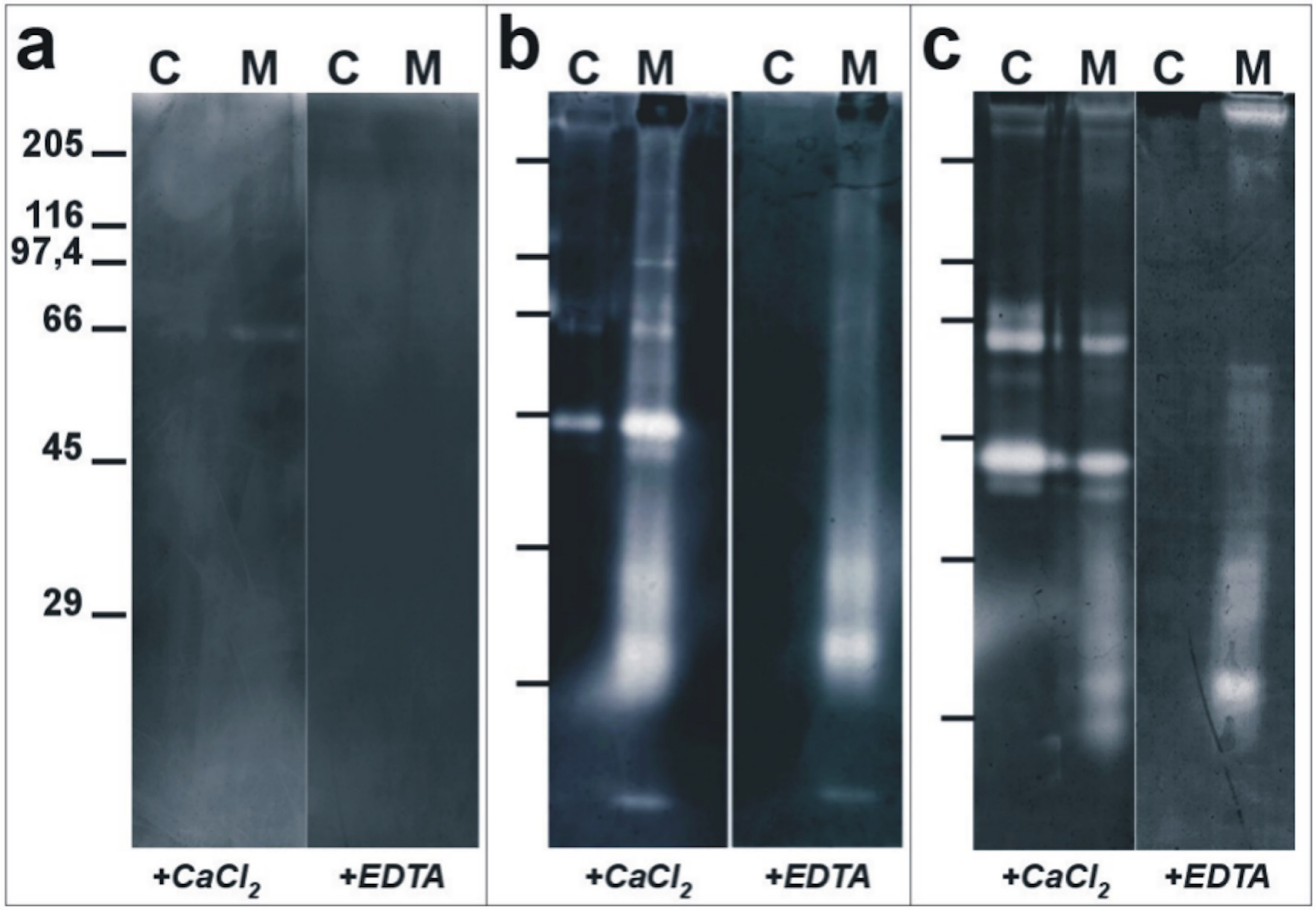
Gelatin zymography analyses of extracts obtained from confluence or migrating endothelial cell bodies, membranes and shed membrane vesicles. Endothelial cell bodies extracts (a), partially purified plasma membranes (b) and shed membrane vesicles (c) were analyzed by gelatin zymography in the presence of 2 mM calcium ions (+ CaCl_2_—activator of MMPs) and/or in the presence of 2 mM EDTA (+ EDTA—inhibitor of MMPs). Extracts were obtained by endothelial cells cultured in conditions in which they reached confluences (C) when stable cell-cell contacts are formed; or from cells that are in active migration (M). Extracts were obtained from cell bodies (a), from partially purified plasma membranes (b) and from shed membrane vesicles (c). Proteins extracted from cell bodies and membranes were loaded at a concentration of 20 μg/well; while, those obtained from shed membrane vesicles were loaded at a concentration of 2 μg/well. Molecular mass markers are reported in kDa.

To compare the expression of proteolytic enzymes in endothelial cells cultured in active migration (M) or at confluence (C), we used gelatin zymographies to test the proteins obtained from cell bodies, membranes and shed membrane vesicles of ECV-304 cells grown in the two experimental conditions. As shown by “Fig 4a”, gelatin zymographies of proteins extracted from cell bodies only show a faint degradative band which, in its electrophoresis mobility, appears to correspond to pro-MMP 2/gelatinase A; in the absence of calcium ions (+EDTA) this lytic band is not observed. Gelatin degradation is more pronounced in extracts of migrating cells compared to confluent ones. Zymographies performed using the same concentration of proteins extracted from the purified plasma membranes of cells cultured at confluence (C) showed three degradative bands, while 13 different degradative bands were detected in extracts of plasma membranes purified from migrating cells (M). When the same samples were subjected to gelatin zymographies in the absence of calcium ions (+EDTA) no proteolytic bands were detected in membranes obtained from cells grown at confluence, while six proteolytic bands were still detected in plasma membranes of migrating cells “Fig 4b”.

Zymographies of proteins extracted from shed membrane vesicles showed a largely increased amount of lytic activity, For these assays, we therefore used only 2 μg of proteins/well in spite of the 20 μg/well used for similar assays in extracts from cell bodies and partially purified plasma membranes. As illustrated by “Fig 4c”, in gelatin zymographies of proteins extracted from membrane vesicles shed by ECV-304 cells cultured at confluence (C), 7 different proteolytic bands are observed, none of which, however, were detected when gelatin zymographies were performed in the absence of calcium ions (+EDTA). Gelatin zymographies of samples obtained from membrane vesicles shed by cells in sparse culture (M) showed 13 bands, 7 of which were also observed when digestion was performed in the absence of calcium ions (+EDTA).

**Fig 5:**
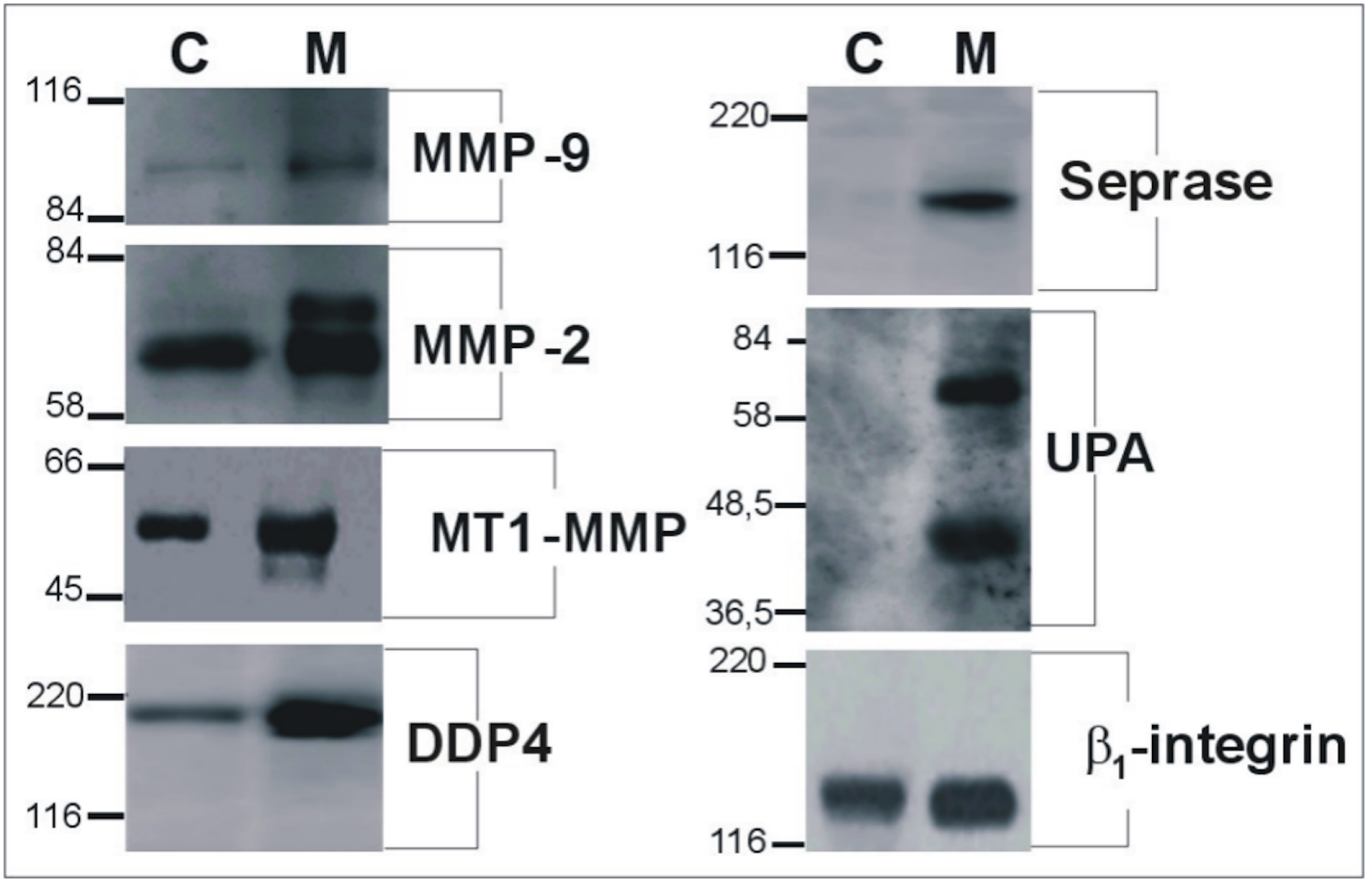
Immunoblotting analyses of extracts obtained from confluence or migrating endothelial shed membrane vesicles and membranes. Immunoblotting assays on shed membrane vesicles obtained from endothelial cells cultured to confluence (C) or in migrating (M) conditions, using different antibodies against different kind of proteases and β_1_-integrin are shown in the figures. On the left of each image, markers are reported in kDa; on the right are specified the antigens.

Proteolytic enzymes expressed in shed membrane vesicles and plasma membranes obtained from endothelial cells cultured in the two conditions described (C) and (M) were also analyzed by immunoblotting assays, “Fig 5”. Extracellular matrix metalloproteases MMP-2 and MMP-9, membrane type metalloproteinase MT1-MMP, and the urokinase type of plasminogen activator (uPA) together the plasma membrane associated serine proteases, seprase (FAPα) and dipeptidylpeptidase 4 (DPP4), were observed in shed membrane vesicles and the expression of all antigens analyzed was found to have increased in membrane vesicles shed by migrating versus confluent cells “Fig 5”.

### Bi-dimensional analyses of proteolytic enzyme by gelatin zymographies performed in different conditions

**Fig 6:**
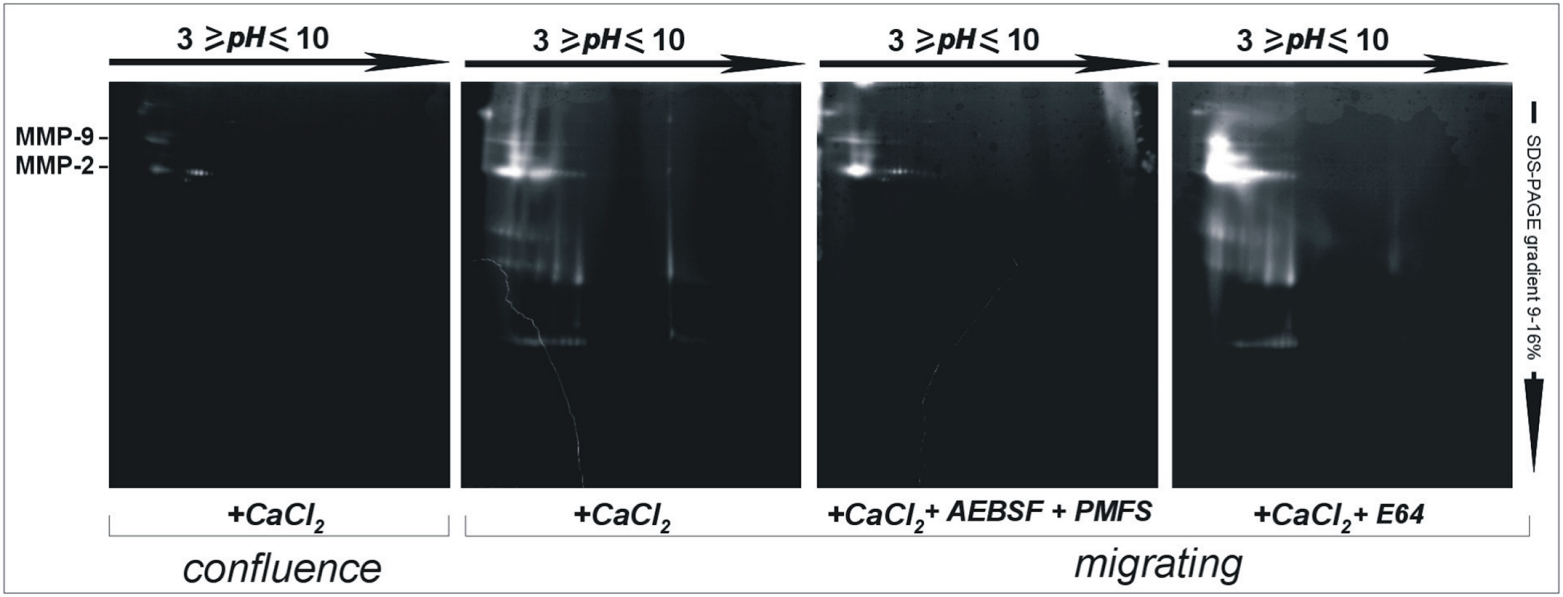
Bidimensional zymography analyses of shed membrane vesicles obtained from confluence and migrating endothelial cell cultures. Shed membrane vesicles obtained by endothelial cells cultured to confluence (*confluence*) or in migrating (*migrating*) conditions were analyzed in bi-dimensional zymography on gelatin substrate. First dimensions were performed on commercial electrophoretical preconstituted strips containing immobilines in a non-continuous pH range from 3 to 10 (Amersham). Second dimensions were performed on SDS-PAGE continuous gradient 9–16% containing gelatin as substrate. Gels were incubated with proteolytic enzyme activators or inhibitors at 37°C for 48 hrs; in the presence of 2mM calcium ions [+CaCl_2_ (activator of MMPs)]; in the presence of 5mM EDTA [+EDTA (inhibitor of MMPs and other proteolytic enzymes regulated in function by bivalent cations)]; in the presence of 2 mM calcium ions plus 25 mM AEBSF and 1 mM PMFS [+CaCl_2_ + AEBSF + PMFS (the last two are inhibitors of serine proteases)]; and in the presence of 2 mM calcium ions plus 1 mM E64 [+CaCl_2_ + E64 (last one is an inhibitor of cysteine proteases)]. Pro-forms of MMP-2 and MMP-9 from human blood plasma were used as markers.

Proteolytic activities present in shed membrane vesicles of endothelial cells cultured in the conditions described (C) and (M) were analyzed by two-dimensional gelatin zymographies. The first dimension was performed on immobiline gel electrophoresis, the second dimension on a 9–16% SDS-PAGE gradient gelatin zymography, as described in Experimental Procedures. All samples were developed in the presence of calcium ions (+CaCl_2_). “Fig 6” shows the large increase in the number of lytic spots evident in gelatin zymographies of membrane vesicles shed by migrating compared to confluent endothelial cells. Extracts of membrane vesicles shed by migrating cells were developed in three different conditions: without protease inhibitors; in the presence of serine-type protease inhibitors 4-(2-aminoethyl) benzenesulfonyl fluoride and phenyl methylsulfonyl fluoride (+CaCl_2_ + AEBSF +PMFS) and in the presence of cysteine-type protease inhibitor E64 (+CaCl_2_ + E64). Lytic activities appeared resistant to E64 inhibition while most bands having low m.w. were not observed when zymographies were developed in the presence of serine-type protease inhibitors.

**Fig 7:**
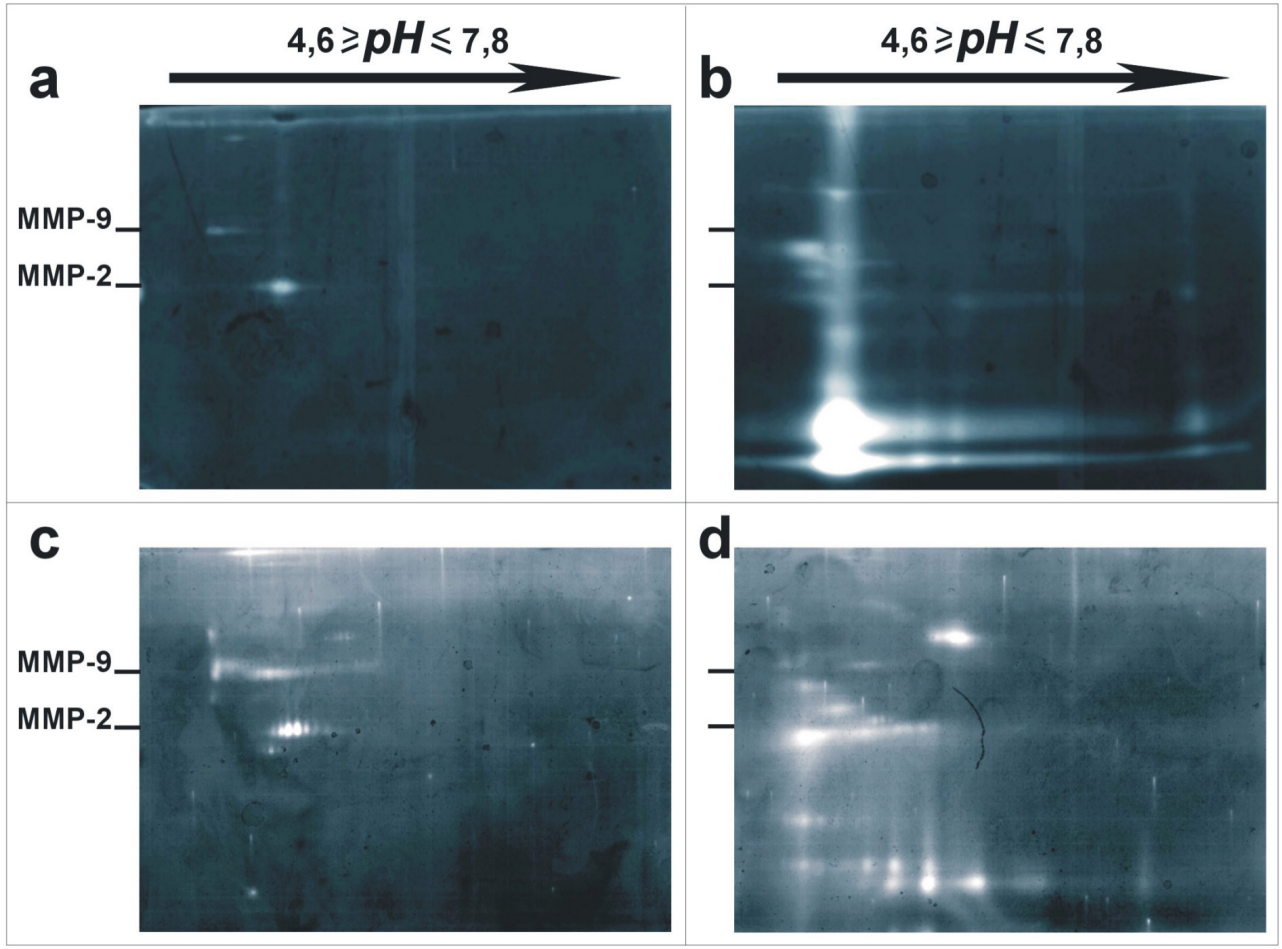
Bidimensional zymography analyses of proteolytic enzymes present in membranes and shed membrane vesicles of endothelial cells. Partially purified plasma membranes (a–b) and shed membrane vesicles (c–d) obtained by endothelial cells cultures to confluence (a–c) or in migrating (b–d) conditions were analyzed in bi-dimensional zymography on gelatin substrate digestion. First dimensions were performed on commercial electrophoretical preconstituted gels in an anpholyte continuous gradient pH range of 4.6 to 7.8 (Bio-Rad). Second dimensions were performed on SDS-PAGE at 7.5%, containing gelatin substrate. Gels were incubated at 37°C for 48 hrs in the presence of 2mM calcium ions (+CaCl_2_). Pro-forms of MMP-2 and MMP-9 from human blood plasma were used as markers.

To better visualize and compare proteolytic activities present in endothelial cell plasma membranes and in shed membrane vesicles, both kinds of extracts were analyzed by bi-dimensional zymographies, in which the first dimension was performed by isoelectricfocusing in a continuous pH gradient ranging between 4.6 and 7.8; and the second dimension on 7.5% SDS-PAGE gelatin zymography. Gelatinolytic patterns of plasma membrane and shed membrane vesicles obtained from endothelial cells cultured to confluence are shown by “Fig 7a and 7c”; those obtained from migrating cells are shown by “Fig 7b and 7d”. The gelatinolytic pattern of shed vesicles appears extremely complex and richer in lytic bands than in vesicles shed by migrating cells.

### ECM component degradative patterns generated by enzymatic activities associated with membrane vesicles shed by endothelial cells

**Fig 8:**
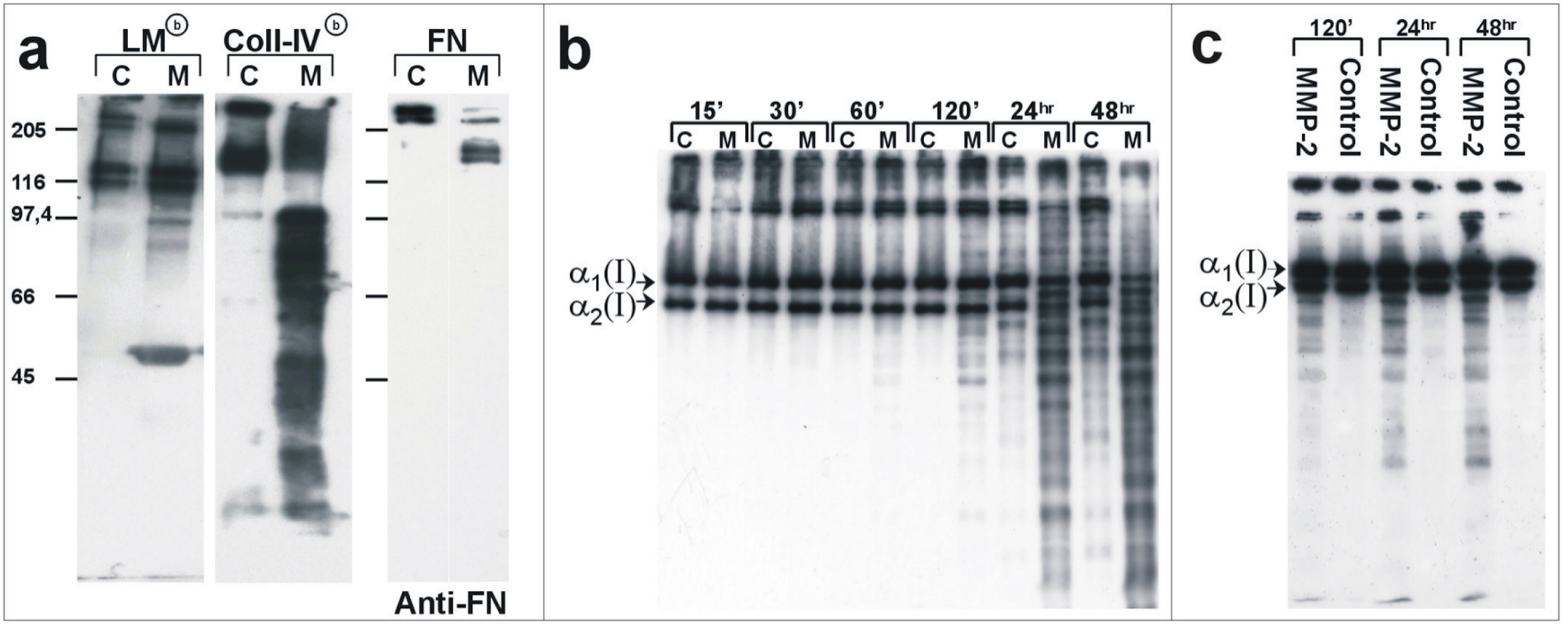
Degradative effects of shed membrane vesicles released by endothelial cells on extra-cellular matrix components. Shed membrane vesicles obtained from endothelial cells cultured to confluence (C) or in migrating (M) conditions were assayed for their capability to degrade different ECM components, in particular they were assayed on basal lamina components, Laminin (LM), Type-IV collagen (Coll-IV) and Fibronectin (FN) (a), and on type-I collagen fibrils (b and c). In (a), we see the degradative patterns obtained by treatment for 24 hrs of basal lamina components. Type-I and IV collagens and Laminin were biotinilated and degradation stained by streptavidin conjugated to HPR and developed with ECL staining (Amersham); while Fibronectin (FN) degradation was stained with mAbs against human Fibronectin. In (b) we see the kinetic degradative patterns obtained by treating jellified type-I collagen with vesicles obtained in different cell culture conditions at different times of incubation. In (c) there are two controls; collagen fibrils treated with MMP-2 originated by molecular engineering (MMP-2) and spontaneous degradation of type-I collagen fibrils (Control) at different times. In (a) markers are in kDa; in (b) and (c) the positions of α_1_(I) and α_2_(I) monomers of interstitial type-I collagen are marked.

Shed membrane vesicles obtained from endothelial cells cultured at confluence (C) and in migratory conditions (M) were analyzed for their capability to degrade basal lamina (Laminin, Collagen type-IV and Fibronectin) and extra cellular matrix (Collagen type-I) components. As shown by “Fig 8a”, proteolytic enzymes associated with membrane vesicles shed by migratory cells digested basal lamina components much more efficiently compared to those associated with membrane vesicles shed by endothelial cells cultured to confluence. Degradative effects of shed membrane vesicles on biotinilated type-I collagen fibrils were assayed after different times of incubation. In samples treated with membrane vesicles shed by migrating cells, degradative peptides were already detected after 60 minutes of incubation; in contrast, an enzymatic degradative pattern was not detectable until 24 hrs after incubation with membrane vesicles shed by endothelial cells cultured to confluence “Fig 8b”. As shows by “Fig 8c”, detected collagen type-I fibrils peptides were neither due to autocatalytical effects (Control) nor to the exclusive enzymatic activity of MMP-2 alone (MMP-2).

### Involvement of proteolytic enzymes associated with shed membrane vesicles in the generation of a permissive substrate for endothelial cell migration

**Fig 9:**
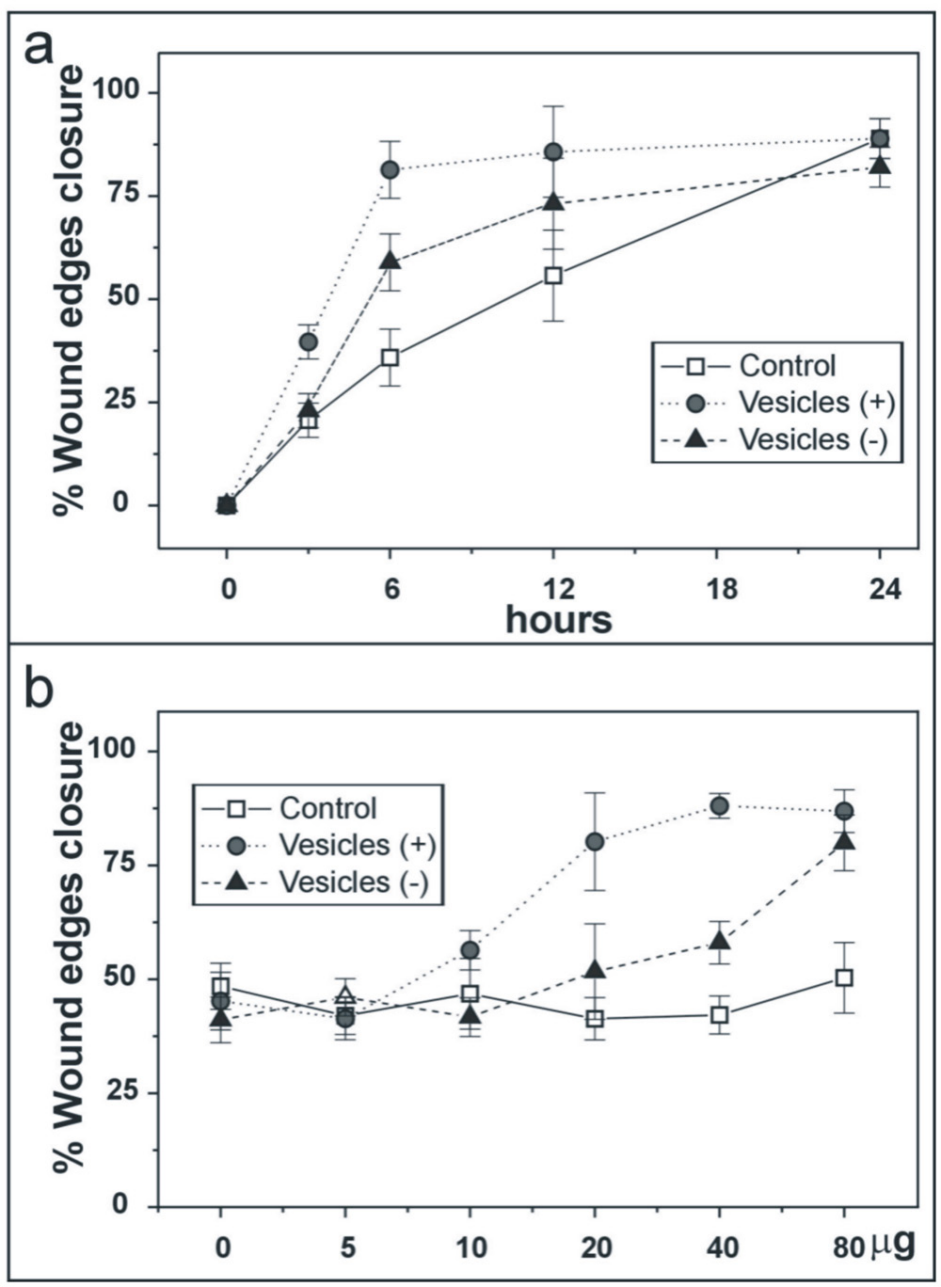
The effects of time-dependent and dose-dependent shed membrane vesicles on the generation of collagen type-I fibril permissive gels to endothelial cell migration. In a monolayer wound closure model on which type-I collagen fibril gels containing shed membrane vesicles produced by endothelial cells cultured in migrating conditions (+) or to confluence (-) were added, endothelial cell migration is quantified as % of wound area closure. In (a) time-dependent wound closure. In (b) vesicles dose-dependent wound closure. Control: No compound was added to the type-I collagen fibril gel seeded on the wounded cells. Vesicles (+): Vesicles generated by endothelial cells cultured in migrating conditions were added to the type-I collagen fibril gel seeded on wounded cells. Vesicles (-): Vesicles generated by endothelial cells cultured to confluence were added to the type-I collagen fibril gel seeded on wounded cells. 40 μg/ml of vesicles were added in (a); in (b) the time of analyses was fixed at 6 hours. The values come from the media of almost three different experiments for each condition. The values are mean ± SD, P value was <0,01 in all analyzed sample.

To analyze the possible involvement of vesicle associated proteases in the generation of a permissive substrate for endothelial cell migration, we performed wound edge assays on type-I collagen fibrils to which shed membrane vesicles had been or had not been added “Fig 9”. Endothelial cell migration was valued as the % of wound edge closure at various intervals following wound insertion “Fig 9a” and after a fixed period (9 hours) from wound insertion, in the presence of different vesicles amounts “Fig 9b”. As shown by the figure, vesicles shed by migrating endothelial cells had a clear stimulatory, dose dependent effect on ECV-304 cell migration. Vesicles shed by confluent endothelial cells had a much less pronounced, but still evident, stimulatory effect on cell migration. These data are supported by migration/invasion assay “S2 File”.

**Fig 10:**
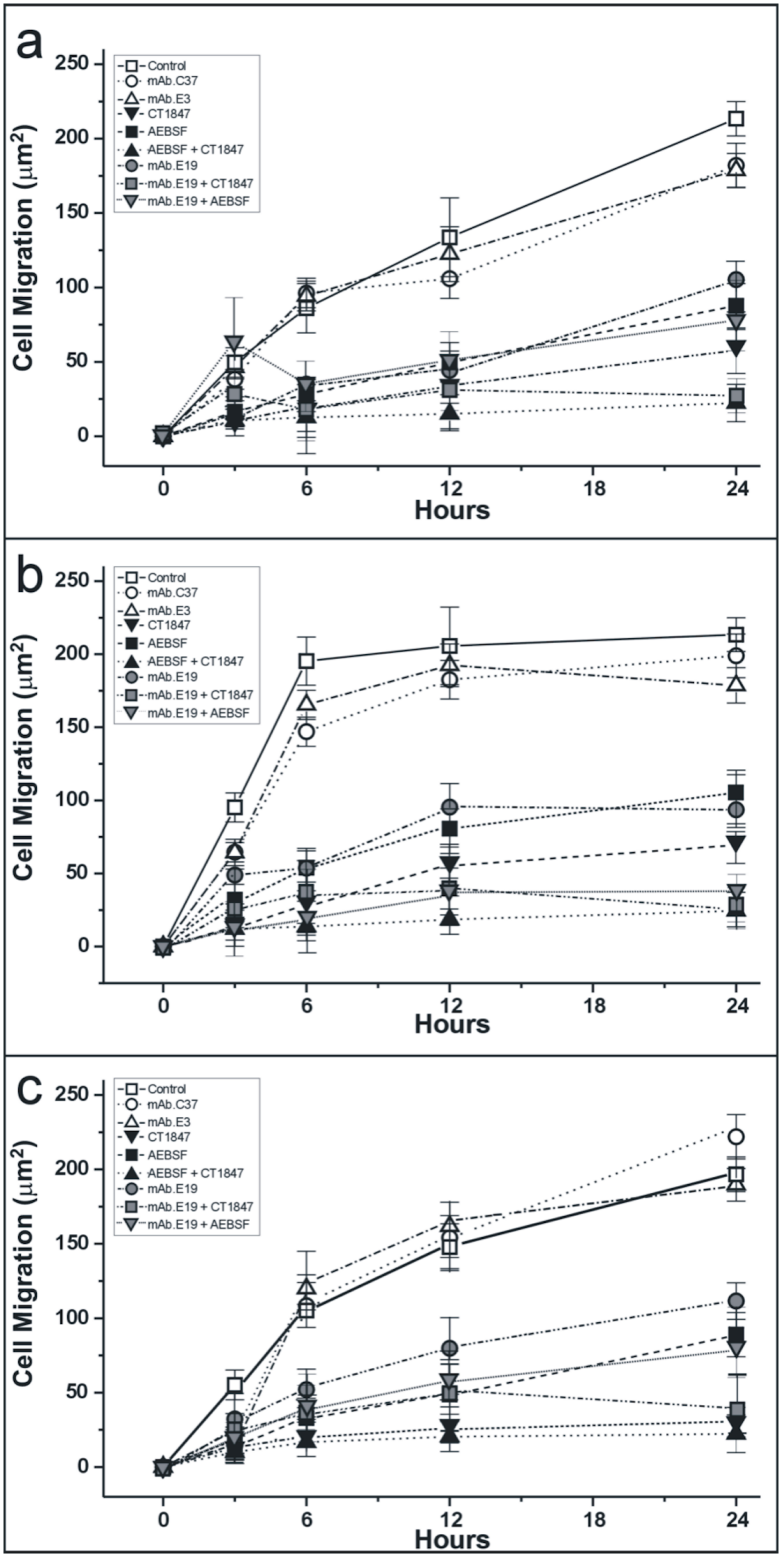
Endothelial cell migration in type-I collagen fibril gels treated and not treated with shed membrane vesicles. (a) Time-dependent endothelial cell migration in-non treated type-I collagen fibrils gels cultured in a monolayer wound closure model in both the presence and absence of proteolytic inhibitors; (b) same conditions as (a) but in the presence of shed membrane vesicles that come from endothelial cells cultured in migrating conditions; (c) shed membrane vesicles coming from endothelial cells cultured to confluence were added to the type-I collagen fibril gels. Experiments were performed in the presence of a specific inhibitor for DPP4 [**mAb E19**], in the presence of a generic serine protease inhibitor [**AEBSF**], in the presence of a generic extra-cellular matrix metalloprotease inhibitor [**CT1847**], and in the presence of a mixture of serine (AEBSF) and extracellular matrix protease (CT1847) inhibitors [**Both inhibitors**]; AEBSF and CT1847 inhibitors were also respectively added to mAb E19 **[mAb E19 + AEBSF] and [mAb E19 + CG184]**, mAb C37 was used as a control against cell surface glycoprotein gp-90, non related to proteolytic enzymes, [**mAb C37**]; mAb E3 against DPP4, while recognizing a non-functional epitope of the molecule [**mAb E3**] and the non-treated cells [**Control**]. The abscissa shows cell migration time [Hours]. In the ordinate, cell migration was quantified by measuring the areas of cell advancements from the original wound edge [Cell Migration (μm^2^)]. Protease inhibitors were added at concentrations of AEBSF 20 μM and CT1847 50 nM respectively; all antibodies, mAbs E19, E3 and C37, were added to 5 μg/ml. Shed membrane vesicles from migrating endothelial cells in (b) and from confluence endothelial cells (c) were added to the type-I collagen fibril gels at 40 μg/ml. Three experiments of monolayer wound models were used for each antibody, inhibitor and control. The values are mean ± SD, P value was <0,01 in all analyzed sample.

To analyze the role of different vesicle-associated proteolytic enzymes in generating the permissive substrate, assays were performed in the absence/presence of different proteolytic enzyme inhibitors “Fig 10”. Endothelial cell migration was assayed in the absence of shed membrane vesicles “Fig 10a”, in the presence of membrane vesicles shed by endothelial cells cultured in migrating conditions “Fig 10b”, and in the presence of membrane vesicles shed by endothelial cells cultured to confluence “Fig 10c”. As shown by the figure, whereas non-functional antibodies (mAb E3 and mAb C37) with an effect on proteolytic enzyme activities had no special effects on cell migration, all enzymatic inhibitors and functional mAb E19 had an evident inhibitory effect on both spontaneous and vesicle stimulated migration of endothelial cells.

## Discussion

In this study, we provide evidence on the clustering of some MMPs, uPA and TTSPs in specialized plasma membrane domains of endothelial cells having a migratory phenotype which in part are released in the extracellular medium as membrane vesicles and in part are also targeted to specific areas of cell membrane where sprouting occurs. The increased expression of proteolytic enzymes in migrating cells and their clustering in specialized domains of the cell plasma membrane was observed by immunolocalization experiments performed in 2D and 3D systems. We noticed that cells invading the wound healing area expressed all the analyzed proteases on their plasma membranes, namely MMP-2, MT1-MMP, Seprase and DPP4. In the same microscopic field, however, most proteolytic enzymes were not detected in cells forming stable cell-cell contacts. Only MT1-MMP was shown to be localized on plasma membranes in both culture conditions. In 3D type-I collagen gel culture system analyzed molecules localized on sprouting plasma membranes as well as in shed membrane vesicles.

The concentration of vesicle-associated proteolytic enzymes evaluated by gelatin zymography or by western-blotting analyses was found to be higher in vesicle membranes in comparison to their main concentration in cell plasma membranes.

In endothelial cells which acquired a migratory phenotype, both the rate of membrane vesicle shedding and the amount of plasma membrane and shed vesicle associated proteolytic enzymes increased significantly. The pattern of gelatinolytic enzyme expression was analyzed by bi-dimensional gelatin zymography. Experiments showed that when endothelial cells acquired a migration phenotype, there was a large increase in the number and intensity of lytic bands detected both in plasma membranes and in shed membrane vesicles. Moreover, the bi-dimensional gelatin zymography assays demonstrated that for some enzymes the presence of several iso-forms in gelatinolytic activity were distributed in a pH range between 5.0 and 7.0; only some of these proteolytic activities were detected in plasma membranes and shed vesicles of cells cultured to confluence. Moreover, experiments performed with E64 inhibitor suggest a not direct involvement of cysteine proteases in endothelial cell motility; also if, we can’t exclude their participation into intracellular maturation pathways during induced angiogenesis in direction of cell motility acquisition from endothelial cells.

On the basis of the relative molecular weight and of the sensitivity to EDTA, the zymography experiments suggest the presence of MMP-2 and MMP-9 in active and pro-forms, and of seprase. The degradation bands to 50/70 kDa not inhibited by EDTA are probably due to cleavage of seprase as has recently been described in tumor cell lines [42]. We also observed a high molecular weight component that could correspond to meprin, and a large lytic band at the top of the gels that, as recently described in migratory endothelial cells [20], corresponds to the high molecular weigh seprase and DPP4 complex. The presence of DPP4, as well as that of MT1-MMP and of uPA was affirmed by immunoblotting experiments performed with specific antibodies.

Clustered proteolytic enzymes expressed by migrating endothelial cells and released as membrane vesicle components are involved in the degradation of ECM and generate a permissive substrate for cell migration/invasion. Shed membrane vesicles were shown to degrade both basal lamina components (Laminin, Type-IV collagen and Fibronectin) and type-I collagen fibrils, which must be viewed as a gelatin, and therefore as a permissive substrate. Vesicles released by migrating cells were shown to be more active in digesting ECM components compared to vesicles obtained from confluence cultured cells; however, both kinds of vesicles exerted degradative effects, suggesting that they play a role in ECM remodeling.

As shown by treatments with proteolytic enzyme inhibitors, the important biological role of membrane vesicles shed by endothelial cells in facilitating cell migration depends on all vesicle-associated proteolytic enzymes. Experiments performed with the aim of identifying proteolytic enzymes which have a key role in this process demonstrated that the proteases involved belong both to the family of metalloproteinase and that of serine proteases and that the two families of proteolytic enzymes have a synergistic effect in the generation of a permissive substrate for endothelial cell migration. Inhibiting either of the two enzyme families causes a decrease in both spontaneous and vesicle-induced cell migration, and a complete block when the synergic effects of both classes of enzyme inhibitors are present. Cell movements are also significantly inhibited when a monoclonal antibody, mAb E19 that specifically inhibits DPP4 activity, is added.

Increased expression of proteolytic enzymes (MMPs and serine proteases) in endothelial cells with a mesenchymal phenotype point to their fundamental role during endothelial cell migration. Moreover, experiments performed on cell migration using inhibitors for the different families of enzyme strongly suggest a synergistic process played from them to generate a permissive substrate.

These data, together those demonstrating the clustering of proteolytic enzymes in specialized plasma membrane domains, make vesicles specific tools in endothelial cell migration/invasion.

## Supporting Information

S1 File ECV-304 cell motility. (PDF)

S2 File Migration/invasion assay. (PDF)
