# Supplementary material for: Proteolytic Enzymes Clustered in Specialized Plasma-Membrane Domains Drive Endothelial Cells’ Migration
Source: PLoS One. 2016 May 6;11(5):e0154709. doi: 10.1371/journal.pone.0154709 (PMC4859482; doi:10.1371/journal.pone.0154709)
Supplement: S1 File — (PDF) [file pone.0154709.s001.pdf]

S[1] Supporting Data

Individual ECV-304 motility was evaluated by time lapse imaging as described in experimental procedures. As shown in Figure *ECV-304 motility evaluation*, cells move in different directions through a media space of 198,75 μm in 17 hrs at a medium speed of 11,63 μm/hr.

Experimentally Endothelial cell motility evaluation was performed as follow: Endothelial cell motility was evaluated as individual cell migration in a partially modified application described by Rosello et al. (2004) [40]. In brief, ECV-304 cells were seeded at very low density in 6 well plates and "time 0" was for the cell migration evaluation was 2 hours after seeding. Twelve cells per microphotograph area (magnification 6.3X) were photographed every hour for 17 hours; the trajectories were plotted on graphs in which the axis origin was considered the start point for each cell. The experiment was repeated six different times; a graph from one of these experiments is included in the results (*ECV-304 motility evaluation*).

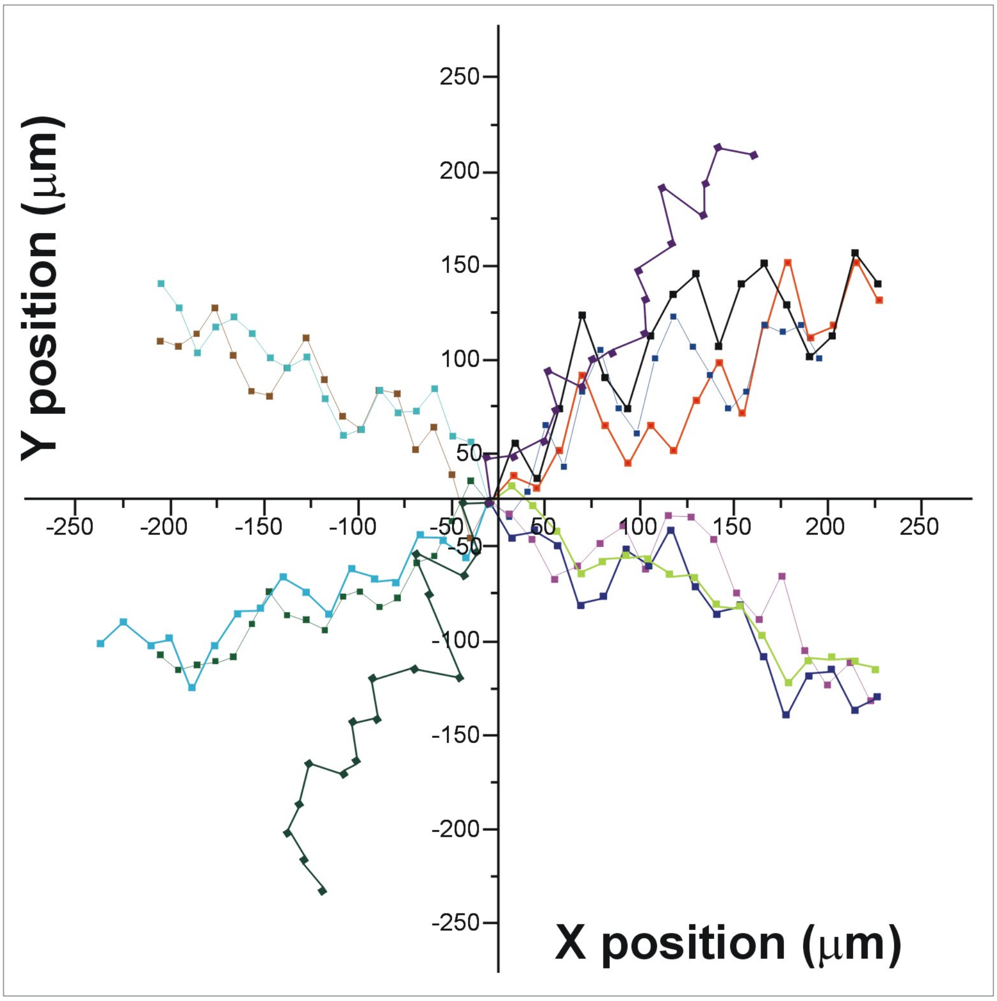

ECV-304 motility evaluation.

Endothelial cells were cultured to very low density. The figure maps the movement of cell positions generated by time lapse imaging analysis of twelve cells in seventeen hours of observation. The starting point for each cell coincides with the axis intersection. The space is reported in μm.
