# Supplementary material for: Proteolytic Enzymes Clustered in Specialized Plasma-Membrane Domains Drive Endothelial Cells’ Migration
Source: PLoS One. 2016 May 6;11(5):e0154709. doi: 10.1371/journal.pone.0154709 (PMC4859482; doi:10.1371/journal.pone.0154709)
Supplement: S2 File — (PDF) [file pone.0154709.s002.pdf]

Support data S2

Migration/Invasion assay

Using the CytoSelect 24 wells Cell Migration and Invasion Assay (8 µm, Colorimetric Format) kit (Cell Biolabs, Inc.), we have test the ECV-304 cells migration in absence or presence of shed membrane vesicles from ECV-304 cells cultured both to confluence that in migrating conditions, as previously described in *Experimental Procedures*. Briefly, into upper chambers of 24 insert plate were stratify a thin gel of collagen type-I (150 µl/insert) and polymerized to 37°C 45'; than were added in each upper well 300 µl of medium containing 10^6 cell plus or minus shed membrane vesicles from confluence or migrating ECV-304 cells (see wound healing test) or without them, as negative control. After 1, 2 and 4 days were performed colorimtric assay of ECV-304 cells that move on the other insert face through the 8 µm hole; colorimetric evaluation of cell migration was perform as reported in data sheet from the vendor.

As shown in Fig. S2, there is an increase in migration of ECV-304 cell when co-cultured in the presence of shed membrane vesicles; in particular in the presence of vesicles coming from migrating cells showing a double amount or more of migrating cells in time compared to the control.

This data is in agreement with other in direction of capability of shed vesicles from migrating cell, and proteolytic enzymes present inside them, to induce a positive remodeling of type-I collagen gel structure.

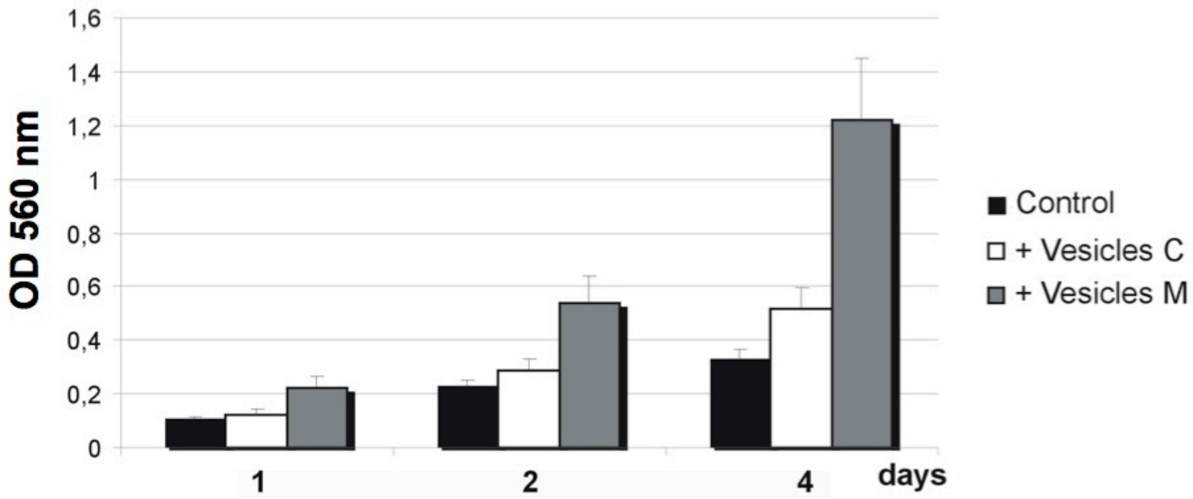

Figure S2 – Colorimetric evaluation of ECV-304 cell migration through 8 mm membrane when cultured in absence (Control) or in presence of vesicles from ECV-304 having epithelial phenotype (+ Vesicles C) and having mesenchymal phenotype (+ Vesicles M) at different days of incubation. Data came from 3 different experiment in which every sample was 8 time represented. The values are mean ± SD, P value was <0,01 in all analyzed sample.
